# (Re)Integrating classical natural history approaches into contemporary field primatology: toward a more holistic understanding of primates

**DOI:** 10.1007/s10329-026-01241-8

**Published:** 2026-02-19

**Authors:** E. Freymann

**Affiliations:** 1 Present Address: Ethnopharmacology and Zoopharmacognosy Junior Research Group, Bernhard Nocht Institute for Tropical Medicine, Hamburg, Germany; 2 Institute at Brown for Environment and Society, Brown University, Providence, RI 02912 USA; 3 Department of Agriculture and Food Sciences, Neubrandenburg University of Applied Sciences, Neubrandenburg, Germany

**Keywords:** Natural history, Primatology, Multidisciplinarity, Inclusivity, Subjectivity, Creative communication, Next-Generation primatology

## Abstract

The historical evolution of the natural sciences, from natural history to specialized scientific disciplines, has fundamentally altered how we study the natural world. While scientific specialization has undoubtedly brought both depth and rigor to specific scientific fields, including primatology, it has also created methodological limitations that fragment our understanding of complex primate ecosystems. This paper outlines four core principles, once central to natural history research, that are less commonly applied in contemporary primatology. These principles include: multidisciplinarity, democratized participation, integration of subjective interpretation, and creative communication. I argue for the strategic integration and/or reintegration of these principles within primatology and provide concrete recommendations for institutional enactment. However, while the incorporation of these selected principles may make the field more inclusive, ethical, and diverse, it is critical to also acknowledge that many other aspects of natural history have been historically rooted in a colonialism, racism, and exploitation. This paper, therefore, does not call for a blind return to natural history approaches as they were once practiced, but rather for the development of a new, holistic, next-generation primatology. Rather than abandoning scientific rigor, this synthesis approach would expand primatology’s methodological toolkit to better reflect the interconnected nature of primate worlds while bridging the gap between academic and local knowledge systems. Such integration offers pathways toward more inclusive, comprehensive, and ultimately more effective primate research and conservation.

## Introduction

Finding a singular and consistent definition of natural history is surprisingly difficult (Barrows et al. 2016; Fleischner et al. 2017; Herman 2002; Schmidly 2005). In this paper, I therefore combine several definitions from various scholars who have written on the topic. Here, I define natural history as a branch of knowledge that embraces the collection, description, and classification of objects in the natural world, and which relies on direct observation for discovery without the prerequisite of a formal hypothesis (Able 2016; Bown 2002; Herman 2002; Tewksbury et al. 2014). Historically, practitioners, known as *naturalists*, ranged from amateur collectors to formally educated scientists (Bown 2002). In addition to their scientific interests, naturalists have also been characterized as having “emotional” and “aesthetic” appreciations for natural phenomenon, which give them credibility with the public (Schmidly 2005; Schmidt 1946).

The formal study of classical natural history dates back as far as Aristotle (384-322_BC_) and the ancient Greco-Roman empires, though human engagement with the natural world long predates this period. Natural history flourished in Europe and America during the 18th and 19th centuries, often described as a “golden age” for Western science (e.g. Bown 2002; Vasile 2018). Colonial expansion and global trade fueled exploration, while new instruments enabled novel methods of observation (Daston and Galison 2007). At the same time, the classification and ordering of the natural world became a cultural project as well as a scientific one, driven as much by curiosity and wonder as by commercial and political interests (Miriti et al. 2023). Naturalists were dispatched to document unfamiliar environments and return with specimens, producing a surge of new knowledge that reached both scholarly and popular audiences.

The flourishing culture of classical natural history was distinguished by its breadth and relative accessibility. Self-taught naturalists, who often had other occupations or were not affiliated with any scientific institution sought to observe the interconnectedness of organisms and environments, often weaving together insights across multiple fields (Bown 2002; Schmidly 2005). Although the field of natural history was by no means universally inclusive, its openness to input from citizen scientists meant that scientific knowledge was both produced and consumed by members of the public. In Europe, the public’s strong interest in natural history was demonstrated through the popularity of scientific narratives, natural-historical travel accounts, and monographs (Bown 2002). Alexander von Humbodlt’s books about his travels to South America, including *Political Essay on the Kingdom of New Spain*, *Personal Narrative of Travels*, and *Kosmos* made him a household name (Rupke 2021). *The Aquarium*, a book published by Gosse (1856) was also widely read, popularizing the observation of marine animals and plants (Brunner 2018). Darwin’s famous *On the Origin of Species* (1859) sold out in just one day (Radford 2008).

In America, public engagement with natural history was also widespread, driven by a growing literate middle class and the early incorporation of natural history into school curricula (Smocovitis et al. 2020). This is reflected in the success of *The American Naturalist* (Dexter 1956), the oldest continuously published biological journal in the United States (Smocovitis et al. 2020). Founded in the late 1860s as a magazine for amateur naturalists and farmers, the journal published personal travel accounts, field anecdotes, engravings, and creative pieces (e.g., musical notations of insect songs) (Hartt 1869; Scudder 1868). Its pages welcomed a diverse range of contributors and readers, capturing the spirit of a field that blurred boundaries between science and culture.

In addition to scientific papers, the complexity of the natural world was also commonly conveyed to the public through the creation of art and scientific illustration. Scientific expeditions during this period often included artists who conveyed the beauty of the natural world alongside its empirical details (Beans 2021; Hood 2023). For example, many of the artworks made on scientific expeditions in the late-18th and19th centuries were influenced by the Romantic artistic movement of the time (Jacobs and Lehman 1995). In addition to rendering botanical, geological, and zoological specimens collected on these journeys, artists often highlighted adventure and beauty in their work, infusing their own emotional responses onto their natural subjects (Hood 2023). Many naturalists were also skilled artists themselves, publishing their own artwork alongside their science. Notable examples include Anna Atkins, John James Audobon, Maria Sibylla Merian, and Ernst Haeckel, to name but a few.

However, by the time the field of primatology formalized as a distinct academic subdiscipline within biology in the mid-20th century (Ankel-Simons 2007), the “golden age” of natural history had long since ended, and many of its integrative practices had been lost to professionalization and specialization (Beer and Lewis 1963). Consequently, the field of primatology, particularly in Europe and the United States, inherited methodological frameworks that emphasized objectivity, technical rigor, and disciplinary boundaries, rather than the observational and participatory approaches characteristic of classical natural history. Revisiting the ethos of classical natural history highlights guiding principles that remain urgently relevant to contemporary primatology, even as the following section traces the gradual disappearance of these principals from the natural sciences over the last century.

### The transformation of scientific practice

According to some scholarship, science’s transition away from natural history began sometime in the mid- to late-19th century as science started to become both professionalized and institutionalized (Beer and Lewis 1963; Smocovitis et al. 2020). By the early-20th century, the accumulated data, observations, and collections created a scale of knowledge that exceeded the capacity of individual researchers (Bown 2002). Simultaneously scientific technologies emerged spurring an era of scientific “technophilia” (Schmidly 2005) which required specialized training, and institutional support. This transformation brought important advances in methodological rigor but also rendered many aspects of natural history research inaccessible to amateurs. Professionalization further marginalized non-academic naturalists by framing their work as non-rigorous or anecdotal (Peters 1980). This shift coincided with the creation of highly specialized academic journals that systematically excluded non-professional scientists from scholarly discourse (Schmidly 2005).

Mathematics also became increasingly integrated into scientific methods, and natural history’s descriptive outputs began to be perceived as subjective in comparison with numerical analyses (Schmidly 2005). Overtime, the naturalists’ methods began to be viewed as soft, speculative, sloppy, and scientifically ineffective (Allen 1979; Peters 1980; Willson and Armesto 2006). Today, the emphasis on objectivity and quantification in the natural sciences is still prevalent, as is the false dichotomy between the social and natural sciences.

Visual and artistic scientific communication has also drastically declined, reducing both research accessibility and researchers’ observational acuity. The practice of incorporating aesthetic, awe-inspiring, and interpreted scientific illustrations in publications, meant to inspire wonder and capture public interest, dissipated in the late-19th and early-20th centuries, in favor of more “objective” mediums (Daston and Galison 1992). The emergence of new camera technologies played a major role in the loss of creative scientific representations, as these photographic techniques became increasingly accepted as a noninterventionist means to capture “objective” truths about the natural world (Daston and Galison 1992; Hood 2023). The scientific (and moralistic) rejection of aesthetic and subjective renderings during this period catalyzed a transition towards “mechanical objectivity” in scientific image-making (Daston and Galison 2007). Overall, the loss of these classical natural history approaches meant that investigations of the natural world became increasingly disciplinized, institutionalized, quantitative, and functional; developments that ultimately limited their ability to capture the complexity of natural systems or engage the attention of the general public.

Contemporary concerns about natural history’s decline have been widespread (e.g. Greene and Losos 1988; Noss 1996; Tewksbury et al. 2014). Many have noted that the naturalists are “dying off” (Noss 1996), and that natural history has been “expelled from the ivory tower” (Dayton and Sala 2001). This decline in institutional and public interest has affected funding for natural history projects and resulted in the loss of irreplaceable expertise. Today, institutional structures supporting such work have largely disappeared (Nanglu et al. 2023; Rohwer et al. 2022), replaced by hyper-specialized disciplines with distinct epistemic cultures. Tewksbury et al. (2014) provide several examples to demonstrate this decline, including the decreasing number of herbaria and natural history museum collections. As a result, historical specimens are becoming increasingly inaccessible for research and are gradually degrading over time (Rohwer et al. 2022). The declining priority of natural history education is also evident in the changing requirements for biology degrees in the United States. In the 1950s, biology majors completed an average of 2.25 courses in natural history, and introductory textbooks were natural history focused. As of ten years ago, most U.S. colleges and universities curricula require no natural history coursework for a biology degree, and the representation of natural history in introductory biology textbooks has decreased by at least 40% (Tewksbury et al. 2014). The number of PhD candidates working towards degrees in natural history-related fields has also rapidly declined (Tewksbury et al. 2014). The gradual disappearance of classical natural history practices provides important context for understanding the epistemic, methodological, and cultural limitations inherited by primatology.

### The state of contemporary primatology

Primatology’s scientific roots are no less entangled with colonial histories than those of classical natural history. From its earliest days, primate natural history research in the 19th and early-20th centuries relied heavily on colonial networks for field access (Haraway 1989). These early studies, conducted primarily by European and American scientists, focused largely on collecting specimens for natural history museums, private collections, and medical research (Haraway 1989; Rodrigues et al. 2022). It was not until the 1930s that the first long-term field primate study was conducted (Carpenter 1934). In recent decades field primatologists, anthropologists, and conservationists, have endeavored to critically identify and dismantle artifacts of our field’s colonial legacy (e.g., Bezanson et al. 2024; Rubis 2020; Waters et al. 2022).

Although primatology is in many ways an historical product of classical natural history—and inherited some of its problematic features—I argue that it simultaneously did not inherit several beneficial elements that once characterized natural historical approaches. Because primatology formalized after classical natural history had already been displaced by increasingly reductionist scientific paradigms, it absorbed many of the constraints associated with this broader shift in scientific practice (Sussman 2011). In the following section, I identify several of these resulting limitations and offer recommendations for addressing these issues by reintroducing four principles drawn from classical natural history.

Before turning to these limitations, however, it is important to recognize that not all primatological traditions diverged from classical natural history to the same degree. Although early primatological research was dominated by North American and European scientists, a robust Japanese primatological tradition emerged in the post–World War II period (Haraway 1989; Langlitz 2020; Riley 2019a), and with it, a new epistemic culture (Knorr-Cetina 1999). By the mid-20th century, Japanese primatology was well established and represented the only major primatological tradition in which researchers conducted long-term field studies within their own country (Asquith 2000; Matsuzawa and McGrew, 2008). Notably, however, Japanese primatologists also engaged in the colonial practices of establishing foreign field sites during this time (Langlitz 2020). Later, between the 1960s and 1980s, additional regional primatological programs developed in primate-range countries such as Brazil, Mexico, China, Vietnam, and India (Bicca-Marques, 2016; Estrada et al., 2006; Rodrigues et al. 2022).

Historically, scholars have often contrasted the epistemic cultures of Japanese and Euro-American (often referred to as “Western”) primatology (Asquith 2000; Haraway 1989; Langlitz 2020; Takasaki 2000). This comparison is of course not absolute but instead provides a useful framework for comparing research traditions within the field (Langlitz 2020). Through such comparisons and ethnographies of scientific practice, researchers have identified important differences in how primatologists have engaged with the natural world (e.g., Asquith 2000; Haraway 1989; Langlitz 2020). One such difference, which is relevant to highlight here, is that Japanese primatology has typically embraced researcher subjectivity (Kutsukake, 2010; Matsuzawa and McGrew, 2008; Takasaki 2000). This view, pioneered by figures such as Kinji Imanishi, is aligned with the ethos of many classical natural historians and contrasts sharply with the contemporary Euro-American emphasis on detached objectivity and quantification (Langlitz 2020). Rather than rejecting the scientific value of subjectivity, Japanese primatology historically treated personal interpretation (including strategic anthropomorphism), as a legitimate and sometimes essential methodological tool capable of generating valuable insights (Takasaki 2000).

I am not suggesting that Euro-American primatology lacks scholars who engage deeply with classical natural history approaches. Indeed, the work of many Euro-American primatologists (e.g., Goodall 1986; Napier and Napier 1985; Sussman et al. 2022) exemplify efforts to incorporate natural historical methods and approaches into their research practices. Rather, my point here is to highlight how classical natural history approaches have filtered unevenly into distinct primatological epistemic cultures, shaped by their own institutional histories, regional ontologies, and scientific priorities. Even as the boundaries of these epistemic cultures bleed together within an increasingly global scientific community (Langlitz 2020), Euro-American academic primatology has remained anchored in its characteristic epistemological frameworks (Asquith 2000). My recommendations below, therefore, may be unevenly directed at Euro-American primatological practices and institutions.

### Four borrowed principles for a next-generation of primatology

While many scholars have declared natural history extinct, others contend that we are living in a moment when the resurrection, renaissance, and reintegration of natural history is not only possible but urgently needed (Dayton and Sala 2001; Nanglu et al. 2023; Tewksbury et al. 2014; Willson and Armesto 2006). Tosa et al. (2021) argue that a new wave of “next-generation natural history” is already emerging, enabled by technological and statistical advances that allow detailed observations to be collected across broad spatial and temporal scales and through citizen-science networks. From this perspective, the practice of observing nature has become more feasible, not less—the only difference being that “natural collections” today often take on new forms. There is also growing interest in revisiting historic natural history materials, including museum and herbarium collections as well as field notes, to extract new data (e.g., Astudillo-Clavijo et al. 2024; Büttner et al. 2022; Teixeira-Costa et al. 2023). This approach, known as the “extended specimen” has already led to some exciting findings (Webster 2017).

Other hopeful scholars have provided actionable recommendations for integrating aspects of natural history into scientific investigations, creating general recommendations for students, researchers, and administrators in the biological sciences (e.g. Nanglu et al. 2023; Tewksbury et al. 2014). The case for reintegrating these principles rests on the fact that such approaches can advance and inform critical areas of science, including human health, food security, and conservation (Tewksbury et al. 2014). Yet, the field of primatology lacks a clear framework for integrating these principles into contemporary research and practice.

I too believe that natural history is not a relic of the past but a living practice that can meaningfully shape our discipline. Primatology need not remain bound to an epistemic culture that feels misaligned; instead, we can move toward a next-generation primatology that integrates natural history’s strengths with contemporary scientific approaches. I argue that many of our field’s current shortcomings can be addressed by reintegrating selected elements of classical natural history. To help propel us in this direction, I identify four principles from natural history that remain highly relevant to primatology today: (1) **multidisciplinary approaches**, (2) **democratized participation**, (3) **acknowledgment of subjectivity and interpretation**, and (4) **creative communication and public engagement**.

Critically, however, advocating for the reintegration of natural history principles first requires reflection on the problematic foundations of the tradition. The field of classical natural history was deeply entangled with colonial, extractivist, violent, and blatantly racist ideologies (Miriti et al. 2023). Many aspects of natural history during the 18th -20th century relied on practices that reinforced hierarchies of culture and race, embedding notions of European superiority within ecological knowledge in ways that continue to influence ecological and conservation research (Miriti et al. 2023). Thus, this proposal should not be mistaken as a call to return to these past practices, nor as an effort to obscure natural history’s troubling legacies. Rather, I argue for a careful recovery of its valuable methodological insights, while explicitly rejecting the colonial frameworks in which they were historically embedded.

### Principle 1: multidisciplinary approaches

**The Problem**: There are very few, if any, standalone primatology departments or graduate programs within European or North American universities. Most primate courses and programs are, therefore, housed within existing Anthropology, Psychology, or Biology departments and as a result remain embedded within those particular disciplines’ established academic silos (e.g., Riley 2019b). Hyper-specialization among primatologists often begins early: undergraduate and graduate programs lack interdisciplinarity, fostering the misleading assumption that primatology can be practiced in isolation. In reality, primate behavior, sociality, ecology, cognition, and evolution are deeply intertwined with the ecological, social, and anthropogenic networks they inhabit (Riley 2013).

In my opinion, one of the largest gaps in foundational primatology training is the lack of skills needed to identify and meaningfully integrate sympatric flora and fauna into primatological research and inquiry. For instance, even though botanical training would greatly enrich analyses of the many plant-related primate behaviors (e.g. feeding ecology, nesting behavior, self-medication, and organic tool-use), most primatologists are provided with no botanical training. This renders many field primatologists unable to recognize (i.e., not primed to see) primate-plant interactions which may have critical social functions or fitness consequences. This limitation can result in grouping plants or plant parts together and assuming uniform adaptive roles in how animals interact with them (e.g., Nishida 1976), overlooking the fact that different species—and even individual plants—can have unique properties and uses (Freymann et al. 2024). A lack of multidisciplinary training may also limit primatologists from noticing, ascribing meaning to, or publishing primate interactions with other sympatric fauna (Freymann et al. 2023; Lee et al. 2021; Pelé et al. 2017), as these multispecies entanglements may fall outside the scope of their primate-focused research questions. Primatologists have long studied how specific ecological conditions (e.g., food availability, rainfall, habitat type) directly influence primate behavior (e.g., Clutton-Brock and Janson 2012; Van Schaik et al. 1989; Wrangham 1980). However, they are often less trained in examining the broader ecological context, including other species and ecosystem interactions, that indirectly shape primate behavior.

In the same vein, despite the fact that climate change and anthropogenic disturbances are rapidly altering primate habitats (e.g., Meijaard et al. 2023; Rothman et al. 2015) and thus impacting primate behavior (e.g., Reynolds et al. 2012) and health (e.g., Gillespie et al. 2005), most researchers are still not taught the basics of climate or conservation science and lack the methodological tools to measure the socio-ecological impacts of these disturbances (though see Riley 2013 for examples of studies on this topic). Relatedly, even though some degree of human–wildlife co-existence is common in sites where primates are studied, few primatologists are trained in the social anthropological methods needed to responsibly assess and navigate human-primate conflicts when they emerge (Hockings et al. 2015; Meijaard et al. 2023). This has prompted the development of ethnoprimatology (Example Box 1), a relatively new subfield which argues for the importance of incorporating anthropological practices into primatology—to better understand multispecies interactions in human-primate contact zones (Fuentes 2010, 2012; Fuentes and Hockings 2010; Malone et al. 2014; Riley 2019a).

While it is unrealistic to expect a single researcher to master all the skills detailed here, there are many steps we can take to expand the field’s capacity to ask broader, more integrative questions and form collaborations with other subfields of the natural sciences. In some cases, these bridges have already been forged (Robbins, 2023). For example, since the 1990s primatologists have been collaborating with geneticists and utilizing genetic analyses to better understand primate behavioral ecology and social relationships (Arandjelovic and Vigilant 2018; Guschanski et al. 2008). However, the interconnected nature of primate ecosystems, referred to by Riley (2019a, b, c) as “zones of entanglement” still demands new approaches which integrate insights from multiple disciplines simultaneously.

**Recommendations**: I am by no means the first to argue that to move toward a more multidisciplinary primatology, we must continue to actively dismantle disciplinary silos and foster structures that support sustained collaborations across fields (e.g., Riley 2019b). Graduate training programs have a central role to play in shaping the next generation of interdisciplinary primate researchers. Primatology students should receive foundational training in adjacent fields (both in the biological and social sciences) and be better prepared to work fluently across disciplinary boundaries. Interdisciplinary mentorship models, where students are co-advised by faculty in different departments, can facilitate this training while modeling collaborative practices. Such approaches are especially critical for research situated in complex ecological or social contexts, where understanding primate behavior may also require knowledge of ecological processes and/or human cultural histories (see Example Box 1). Cultivating anthropological skills such as cultural sensitivity, particularly for international researchers working in primate habitat countries, is paramount (e.g., Rubis 2020), as is critical analysis of how the cultural contexts in which we work impact our research (Riley 2019a; Waters et al. 2022). Equally important as learning interdisciplinary methods is training in interdisciplinary ethics and best-practice protocols so that research meets standards across all fields it engages (see Badihi et al. 2026; Bezanson and McNamara 2019; MacKinnon et al. 2014; Riley 2019bb; Riley & Bezanson, 2018; Fedigan 2010).

For this shift to occur, institutional structures must evolve accordingly. Universities will need to create tenure-track positions explicitly designed for interdisciplinary researchers whose work does not align neatly with the traditional departmental categories historically associated with primatology. Promotion and hiring criteria will, therefore, need to adapt to recognize the value of co-authored, collaborative work, which is still under-valued by hiring bodies and avoided by early-career academics for fear it “dilutes” scholarly credit (Kiermer 2023). Search committees should also take into consideration that multidisciplinary studies can be harder to publish as there are fewer peer-reviewed journal options for this kind of research. When it is possible to find an appropriate journal, these publications may not necessarily have as high-impact factors as more disciplinary journals. In sum, building a more integrated primatology will require coordinated shifts in training, institutional structure, and scholarly culture. Without these changes, early-career primatologists who engage in multidisciplinary work risk being undervalued in systems that still privilege disciplinary rigidity (Baker 2024).

Example box 1: ethnoprimatology as an interdisciplinary challenge to disciplinary silos Ethnoprimatology, a subfield of primatology that emerged in the early 2010s, is both a conceptual framework and methodological orientation for studying primates that explicitly situates humans as integral components of primate ecosystems (see Fuentes 2012; Fuentes and Hockings 2010; Malone et al. 2014; Jones-Engel et al. 2003). Ethnoprimatology’ manifesto rests on three key principles: (1) humans and other primates have long interacted across diverse ecological and cultural interfaces; (2) rapid human-driven niche construction has transformed ecosystems in the Anthropocene, making it impossible to study primates apart from anthropogenic ecologies; and (3) therefore, primatology must adopt inherently **multidisciplinary approaches** (Fuentes 2012).

In practice, ethnoprimatology necessitates a diverse methodological toolkit, combining field primatology, behavioral ecology, ethnography, human ecology, history, geography, and even folklore studies (Fuentes 2012). This integrative approach requires collaborative research teams spanning multiple disciplines and knowledge systems (e.g., Fuentes 2010; Rubis 2020). Although still a relatively young subfield, ethnoprimatology has challenged the long-standing siloed structure of primatology by reframing humans and nonhuman primates as co-participants in shared ecologies and evolutionary trajectories, while demonstrating the necessity of anthropological methods for disentangling these relationships.

### Principle 2: democratized participation

**The Problem**: Science currently operates on a foundation of exclusion, and primatology is no exception (Hobaiter et al. 2021). A core tenet of classical natural history, though it was by no means equitable and operated within the classist, racist, and sexist norms of its time, was the inclusion of non-professional naturalists operating outside of traditional institutions, many of whom were self-taught. These “amateur” scientists brought new skills and perspectives to the natural sciences which often led to novel and unexpected findings. Today, however, one of the most persistent barriers to democratized participation in primatology lies in the structural marginalization of non-academic expertise. This most obviously manifests itself in the exclusion of voices and perspectives of people from primate-range countries. For example, primate field station staff, often referred to as “field assistants”, are often unfairly credited only as data collectors rather than as expert observers, conceptualizers, mentors, or co-authors (Covert 2019). Despite the fact that primate field staff often possess deep wells of observational knowledge, accumulated through lived proximity to primate populations (Taylor 2023), institutional norms and dominant scientific centers (which remain largely Euro-American) continue to privilege technical training and formal academic credentials over experiential insight. The exclusion of habitat-country researchers and field staff from primatological discourse serves to further perpetuate neo-colonial practices of ‘parachute-science’ by international researchers (Hobaiter et al. 2021). Cumulatively, these hegemonic structures create and maintain hierarchies of legitimacy within academic primatology regarding whose primatological expertise is considered more scientifically valuable (Asquith 2000).

**Recommendations**: To increase inclusivity in next-generation primatology, it is imperative that our field shifts toward more equitable, community-centered research partnerships. All collaborative projects led by non-habitat country researchers, for example, must be designed from the outset with shared authorship models that recognize habitat-country participants as co-researchers regardless of academic affiliation (Setchell et al. 2025). All too often in our field, habitat-country field staff and researchers are still left uncredited, despite contributing substantial, scientific work (e.g., data collection, teaching students, designing study methods, and developing hypotheses).

Critically, new journals and alternative platforms must also be created which accept and disseminate scientific and/or observational outputs related to primatology that center local ecological knowledge and traditional knowledge systems. While in some cases, these outputs may not meet the current criteria or formats of academic peer-reviewed journals, they have already been shown to add new and important perspectives which can benefit primate research and conservation (see Example Box 2) (Taylor 2023).

Example box 2: the perspectives collective journal as a model for democratized participation in primatology The Perspectives Collective Journal *(*https://www.perspectivescollectivejournal.com) is a multimedia online platform that demonstrates how primatology can embrace democratized participation outside of academic institutions. By integrating text, images, audio, and video, it amplifies diverse voices, fosters multilingual storytelling, and centers lived experience alongside traditional research findings (Taylor 2023). The journal, managed by field staff at the Budongo Conservation Field Station (BCFS), a chimpanzee research station in Western Uganda, aims to demonstrate both the feasibility and benefits of this new model of primatology. By amplifying diverse voices, fostering multilingual storytelling, and centering lived experience, such efforts show how the field can evolve into one that is not only more inclusive but also more insightful. For example, in a video interview featured on the platform, Muhumuza (2024) explores how clan totems (animals traditionally protected in Uganda), can influence primate conservation and local stewardship of primate-habitats across different regions of the country.

### Principle 3: acknowledgement of subjectivity and interpretation

**The Problem**: Contemporary primatology, particularly in North America and Europe, like many of the sub-fields nested within the natural sciences, still directly equates rigor with quantification and alleged objectivity (Riley 2019a). Early critiques of anthropomorphism within academic primatology, dating back to critiques of Jane Goodall’s early work in Gombe (Goodall in Bekoff 2007; Riley 2019a), reinforced the expectation within Euro-American primatology that researchers should treat their subjects as objectively as possible. By the 1970s, a shift toward quantification had taken place within the field, and with it a rejection of subjectivity (Strier 2003). This created an epistemic culture in which interpretive insights, observational anecdotes, and researchers’ emotional responses are generally treated as sources of bias rather than as legitimate contributions to understanding (Vitale and Pollo, 2011). Today, the majority of high-impact primatological studies are hypothesis-driven (Strier 2003), use an a priori approach (Kappeler and van Schaik 2002), and rely on statistical modelling (Langlitz 2020). The assumption underlying the preferential use of these methods is that numerical data is best suited to capture the nuance of social relationships, emotional dynamics, and ecological contexts. The issue is: many of these quantitative methods are still rooted in subjectivity, just not in transparent ways.

The objectivity of statistics, for example, is increasingly being called into question as several studies have pointed out that the statistical analyses being used across the natural sciences are often misrepresentative and biased toward false-discoveries (Smaldino and McElreath 2016). Even when data are subjected to unbiased statistical analysis (if this exists), they remain the product of a data collection process that hinges on translation: behaviors are observed, interpreted, and consciously categorized into data points through the subjective selection of particular behavioral ethograms (Langlitz 2020). In field primatology, data collection is also often collaborative, involving input from field staff colleagues, thereby adding layers of (often multi-cultural) interpretive exchange. Even the habituation process (a common prerequisite for establishing a primate field site) is an intersubjective process in which humans and primates co-adapt and negotiate trust over time (Riley 2019a). The data we collect, the questions we choose to ask, the meanings we assign to our observations, and the analytical frameworks we employ are all, therefore, filtered through our lenses and positionalities.

By denying subjectivity in primate research, we obscure the fact that researchers inevitably bring distinct worldviews to their work and that these worldviews change their interpretations of primate behavior (Riley 2013, 2019a, c). Sun et al. (2020) compare primatology to quantum research, contending that objectivity emerges not from eliminating subjectivity but from accounting for it. To deny this intersubjectivity under the guise of quantification is to misrepresent the complexity of the process. Rather than being a disadvantage, Dayton and Sala (2001) saliently note that our human subjectivities (e.g. interpretations, empathy, and sensitivity to natural patterns) should be considered *central* to our primatological insight. These unique positionalities, when acknowledged, allow us to synthesize perceptual experiences into new testable hypotheses (2019). Recognizing these influences allows for more honest, accountable, and ultimately rigorous science.

**Recommendations:** Primatology can benefit greatly from integrating greater reflexivity into research design and reporting, following models established in social anthropology and other social sciences (Riley 2013; Rodrigues et al. 2022; Setchell et al. 2025). Incorporating reflexivity into our practices is not meant to make the field more “soft” or less rigorous. To the contrary, when done appropriately, I echo others in arguing that it will make our field even more rigorous and importantly—more honest. Such reflexivity involves acknowledging how a researcher’s background, beliefs, and social positioning shape their questions, field interactions, and interpretations (Fuentes 2011; Riley 2019a). To facilitate this, graduate training programs should prepare students to work with their interpretive biases, teaching them to recognize how sociocultural frameworks shape both observations and methodological choices (Riley 2019a; Strier 1994). Research protocols should also strive to re-incorporate qualitative observations as legitimate forms of data, especially when they generate hypotheses or reveal patterns invisible to purely quantitative approaches.

Institutional norms must also evolve to support interpretive work. To enact this, scientific journals must develop evaluation frameworks that can accommodate manuscripts which incorporate and/or acknowledge qualitative insights, including wider acceptance of observational studies, while funding bodies should encourage project proposals which explicitly address subjective biases. Finally, we must collectively strive to cultivate *epistemic humility* (Matthews 2006): acknowledging that no single perspective fully captures the realities of primate worlds. This epistemic humility will also assist in the incorporation of both Principles 1 and 2, as we forge new scientific collaborations with people from other academic fields and cultural backgrounds. As Rodrigues and colleagues (2022) argue: “At the interpersonal level, effective and respectful communication is facilitated by understanding each other’s positionality.”

Example box 3: Japanese primatology as a model for effective subjectivity in science Japanese primatology’s epistemic legacy of acknowledging subjectivity provides a compelling example of how the incorporation of this principal has enhanced scientific insight. Unlike historic Euro-American primatological traditions, which have emphasized objectivity and the avoidance of anthropomorphism, Japanese primatologists (many trained at the Kyoto School) have long embraced careful individual identification, close observation, descriptive methods, and the attribution of motives to primate behavior (e.g., Asquith 1996; Langlitz 2020; Takasaki 2000). By engaging empathetically with study subjects and accepting the influence of the researcher’s presence, researchers trained in Japanese primatological traditions repeatedly identified key features of primate social life well before their Euro-American counterparts (Asquith 1996), including socially negotiated dominance (Itani 1961), individual personalities (Imanishi 1960), and social transmission (Kawai 1965). These early discoveries have been attributed in large part to Japanese primatology’s epistemic culture of embracing subjectivity, individual recognition, and interpretive description (Langlitz 2020; Riley 2019a; de Waal 2003).

### Principle 4: creative communication and public engagement

**The Problem**: Primatology today faces an accessibility problem: valuable scientific research is being produced, but it is not necessarily reaching the relevant audiences or stakeholders. Part of the reason for this inaccessibility is the lack of creative approaches to translating densely academic research for popular audiences. Today, creative and visual tools are often under-valued and under-utilized in primatology. Scientific publications frequently rely on dense text and technical jargon, which limit understandability for non-specialists (Martínez and Mammola 2021). Creative outputs (such as narratives, photographs, comics, poems, games, illustrations, films, and field sketches) are essential to effective communication, not only for conveying facts and findings, but also for evoking a sense of wonder and engagement that sustains interest and contextualizes the study’s purpose (e.g., Bezanson 2021; Dahlstrom 2014).

Beyond science communication, the erosion of creative documentation skills has also diminished observational acuity. When researchers sketch, diagram, or visually record primate behavior and habitats, regardless of their artistic skillset, they often notice subtle interactions, behavioral cues, and environmental patterns that might be missed in purely textual or numerical records. Field sketches can even capture valuable data that may be of value decades later (Astudillo-Clavijo et al. 2024). Incorporating visual methods into fieldwork can encourage primatologists to slow down, engage more deeply with their study subjects, and reflect on the ecological and social context of behavior, which in turn fosters both rigor and holistic thinking in the field. By reinvigorating visual communication as a core research practice, therefore, primatologists can improve both the effectiveness of their conservation messaging, and the quality of their scientific observations.

**Recommendations**: In next-generation primatology, creative notetaking and communication approaches should be incorporated as a standard component of field protocols, with researchers receiving training in either scientific illustration or multimedia documentation to enhance public engagement and sharpen observational acuity. Graduate programs should include coursework on visual communication techniques and effective scientific storytelling to prepare future researchers for broader outreach. Publication formats and journals should similarly evolve to embrace visual elements alongside traditional text, ensuring rigorous science is accessible and compelling to more general audiences. As part of this aim, community-based collaborations can ensure local artistic traditions and communication styles are incorporated into creative outputs, enriching both scientific and context-specific cultural understanding.

On an institutional level, dedicated funding and support are needed for creative communication initiatives. These could include training primatologists in creative storytelling, establishing artist-in-residence positions at field stations, and dedicating paid opportunities within primatology research centers and labs for science communicators. Supporting these initiatives will ensure primate research reaches broad, diverse audiences critical to conservation efforts.

**Fig. 1:**
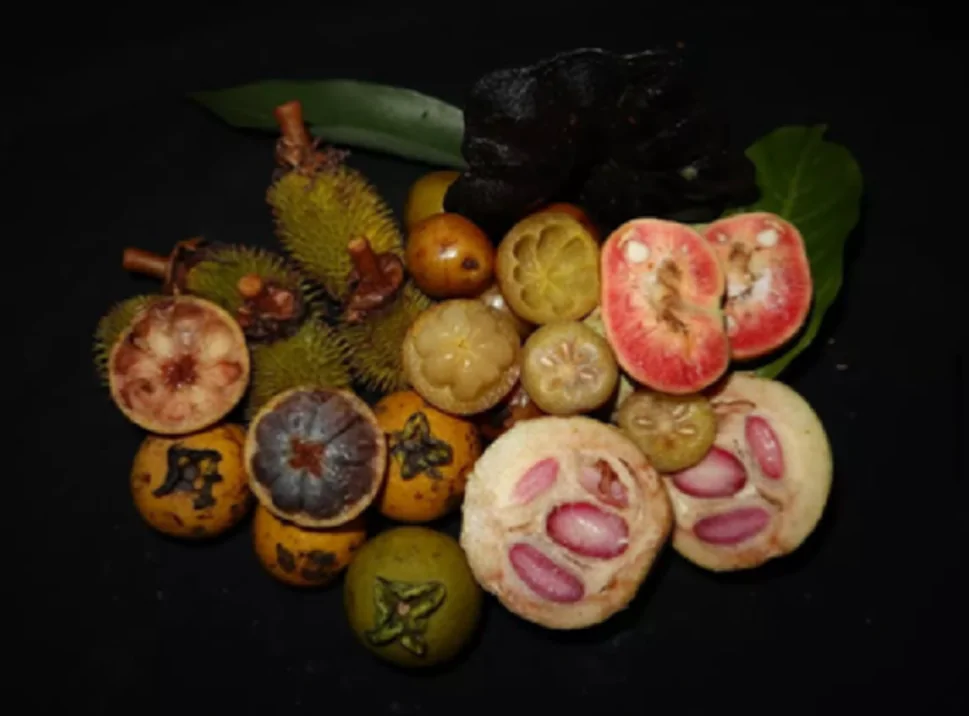
Composite of orangutan seasonal diet from the Gunung Palung National Park, August 2023

**Fig. 2:**
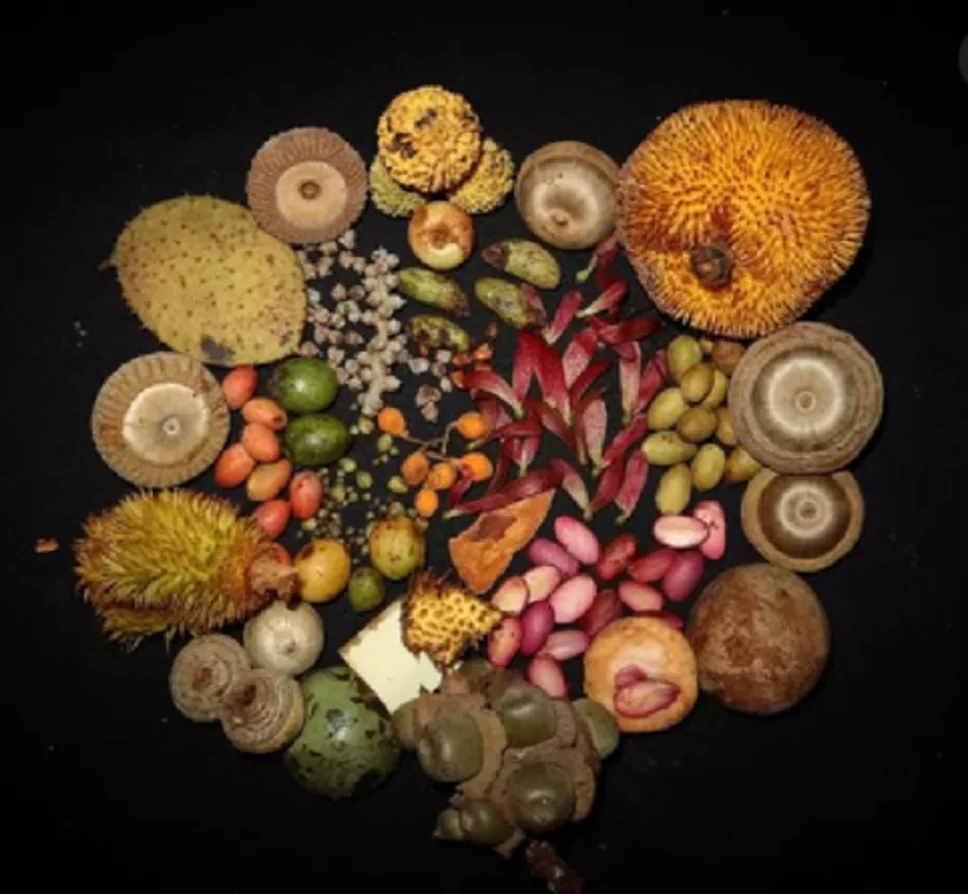
Composite of orangutan diet from the Gunung Palung National Park, December 2023 Image Credit: The Instagram account of Sumihadi (@sumihadi_)

Example box 4: integration of creative visuals as scientific practice A contemporary example of art as a scientific tool in primatology can be seen in the work of Sumihadi, whose Instagram account (@sumihadi_) documents orangutan diets within the Gunung Palung National Park in Borneo. Sumihadi, who works at the site, collects and arranges foods in the orangutans’ monthly diet in ways that reflects both scientific rigor and artistic sensibility (Figs. 1 and 2). By combining detailed field observation with visually compelling storytelling, Sumihadi reaches diverse online audiences beyond academia, illustrating how visual communication can serve both as methodology and outreach. These monthly collections demonstrate that art and science, far from being opposites, can enhance one another and extend primatology’s reach into public consciousness.

### Implications for primate conservation

As of 2025, approximately 68% of primate species are at risk of extinction, with 93% of species experiencing population declines (Estrada et al. 2025). These figures make it painfully clear that our field needs a more effective strategy for protecting primates and their habitats, before time runs out. I argue that reintegrating natural history approaches into modern primatology should be a component of this strategy. The principles outlined above not only strengthen primate research but also enhance conservation outcomes by improving scientific rigor and deepening public engagement.

Conservation of primate habitats and landscape restoration can be greatly enhanced by incorporating detailed natural history data, which often requires multidisciplinary collaboration among primatologists, conservation biologists, and land managers. Tewksbury et al. (2014), for example, document multiple cases in which natural history observations, such as species-specific feeding preferences, seed dispersal patterns, and habitat use, directly informed forest management plans, leading to improved regeneration of native tree species and increased habitat suitability for wildlife. Integrating natural history observations into conservation policy through the inclusion of local and Indigenous knowledge further ensures that strategies are grounded in a nuanced understanding of both ecological and social contexts (Waters et al. 2022). For instance, participatory research in northern Vietnam has drawn on the local ecological knowledge of hunters in the Ngoc Son Ngo Luong Nature Reserve to document the status, historical distribution, and decline of gibbons, langurs, and other threatened vertebrates within a key biodiversity corridor. This has provided baseline data for conservation planning and management. By including long-term natural history observations from local hunters in the absence of historical records, such approaches can inform decision-making processes and conservation plans in ways that would not have been possible through conventional survey methods (Cano and Tellería 2013).

Studies on great ape cognition and welfare also demonstrate that empathy and emotional engagement, such as giving individuals names or strategic anthropomorphizing, can alter researchers’ moral considerations leading to improved care practices and/or catalyzing involvement in conservation initiatives (Vitale and Pollo, 2011). Likewise, acknowledging emotional investment throughout the research process can also help researchers translate complex findings for non-scientific audiences, which may also lead to primate welfare or conservation action (Naguib et al. 2023; Waal and de Waal 2007). At a time when the planet and its biodiversity face escalating existential threats, this is not the moment to retreat behind a façade of detached objectivity or to deny the role that values and emotions play in shaping scientific inquiry. Rather, these affective dimensions should be recognized as integral to science itself, influencing the questions we ask, the methods we employ, the collaborators we engage, and the ways in which we communicate our findings.

Lastly, natural history’s emphasis on creative and accessible modes of communication offers powerful opportunities to cultivate emotional connections with primates and the ecosystems they inhabit. Visual media and creative storytelling can convey the significance of conservation challenges in ways that purely technical presentations often cannot, fostering empathy and motivating action. When research inspires both wonder and emotional engagement, it lays the groundwork for sustained public support, an essential component of long-term conservation success.

## Conclusion

In this paper I have argued that the future of primatology may benefit from recovering certain approaches and methodological traditions from classical natural history. This does not mean abandoning advances in rigor and theoretical sophistication which characterize contemporary research today. Rather, it means balancing these strengths with the interdisciplinarity, inclusivity, subjectivity, and creativity which used to be integrated into the study of the natural world. This revival, which I call next-generation primatology, is not meant to represent a return to outdated approaches, but rather the recovery of valuable traditions which can enhance the relevance, accessibility, and effectiveness of contemporary primate research and conservation.
